# Polymicrobial Cardiac Implantable Electronic Device Endocarditis in a Patient With Device Expulsion

**DOI:** 10.7759/cureus.108441

**Published:** 2026-05-07

**Authors:** Luke Johnson, Navneet Gupta, Ryan Rothman, Michelle Paulson

**Affiliations:** 1 Department of Internal Medicine, Allegheny Health Network, Pittsburgh, USA; 2 Department of Epidemiology and Public Health, University College London, London, GBR; 3 Division of Infectious Diseases, Allegheny Health Network, Pittsburgh, USA

**Keywords:** angiovac and infective endocarditis, antibiotic-resistant bacteria, antibiotic selection, bacterial endocarditis, device-associated endocarditis

## Abstract

Cardiac implantable electronic device (CIED) lead erosion and device expulsion are rare complications that are associated with significant morbidity and mortality. We describe the case of a 74-year-old man who presented with fever, chills, and night sweats after spontaneous implantable cardiac defibrillator expulsion over a decade after its implantation. A transthoracic echocardiogram revealed a large intracardiac vegetation, and a combination of blood and hardware cultures identified *Staphylococcus hominis*, *Corynebacterium striatum*, and *Citrobacter koseri*. Management involved percutaneous vegetation removal, device extraction, pocket debridement, and a prolonged course of targeted antibiotics. This case highlights the potential for very late device erosion and subsequent endocarditis, via either direct microbial seeding of intracardiac structures, hematogenous spread from the pocket infection, or both. It underscores the importance of early recognition, blood and device cultures, and timely device extraction in managing CIED-related infections.

## Introduction

Cardiac implantable electronic devices (CIEDs) include permanent pacemakers (PPMs), implantable cardiac defibrillators (ICDs), and biventricular devices for cardiac resynchronization therapy (CRT). CIED lead or pocket erosion - the gradual breakdown of the skin overlying the lead or device - is a relatively rare complication that can result in valvular or lead infections, with reports of it occurring in between 0.3 to 3% of patients [1,2]. These infections are associated with high morbidity and mortality. Typically, these occur within the first month following device implantation, though later cases have also been described [3]. Risk factors for lead or pocket erosion include lack of subcutaneous fat [4], multiple device revisions [1], and superficial device placement [5]. Management typically involves treating the local site and excluding systemic infections, which require device or lead extraction and replacement [6]. Device expulsion, where part or all of the device is physically ejected out of the body following erosion through the skin, is an extreme manifestation of pocket erosion which leaves a direct anatomical sinus tract along the pathway from the non-sterile skin surface to the intracardiac structures via the device’s leads. This sinus tract can potentially facilitate the translocation of bacteria from the skin directly to the heart. Device expulsion is extremely rare; we have only identified one similar published case [7]. We present a case report of an ICD that was spontaneously expelled from a patient over a decade after its initial implantation. This resulted in *Staphylococcus hominis*, *Citrobacter koseri*, and *Corynebacterium striatum* endocarditis, which required vegetation removal, extraction of the remaining lead, pocket revision, and a prolonged course of antibiotic therapy.

## Case presentation

A 74-year-old gentleman with a past medical history notable for coronary artery disease requiring coronary artery bypass grafts and heart failure with reduced ejection fraction was referred to our hospital by his outpatient cardiologist in view of a worsening wound at his ICD lead insertion site.

The patient had a ventricular pacing and sensing inhibit (VVI) ICD inserted over a decade ago for primary prevention of sudden cardiac death given his heart failure with reduced ejection fracture (ejection fraction 21% at that time) and New York Heart Association (NYHA) class II symptoms. Since the time of insertion, our patient had undergone frequent device checks and reported no history of any symptomatic tachyarrhythmias or ICD shocks. He had a routine generator change undertaken six years prior to replace the device’s battery. This occurred without complication.

Three weeks prior to presentation, our patient noticed a skin erosion and increased pressure in the left upper chest region, following which the ICD generator and distal lead became exposed and expelled from the insertion pocket. He removed the ICD generator by cutting the exposed wire with a garden cutter, after which he developed fevers, chills, and night sweats. He was evaluated in his local emergency department for an erythematous, purulent wound in the left upper chest region; topical bacitracin was prescribed and local wound care advised. He followed up with his local cardiologist after which he was referred to our hospital with high concern for endocarditis given his systemic symptoms in the context of recent device expulsion.

**Figure 1:**
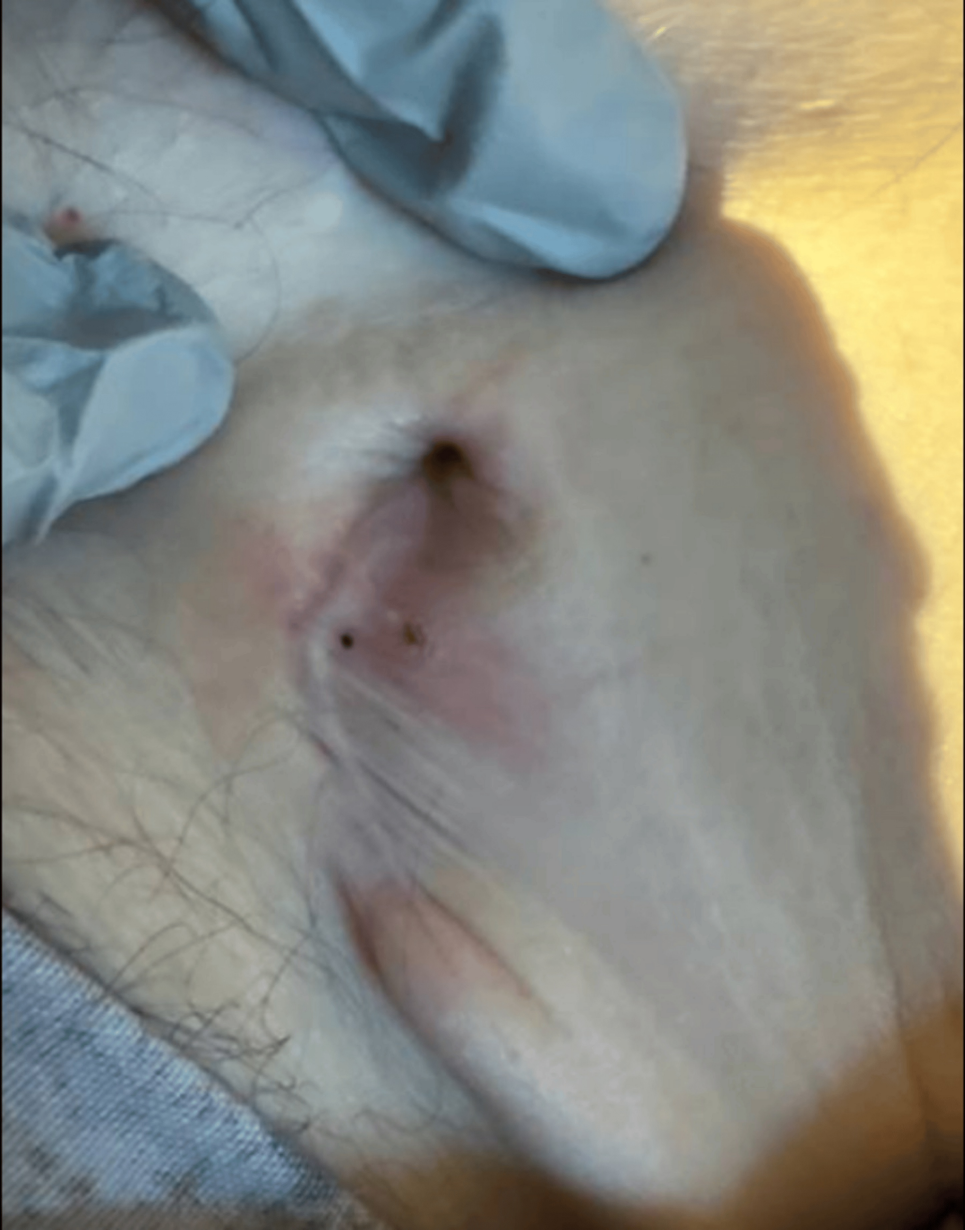
Photograph of the healed wound on the left upper chest out of which the implantable cardiac defibrillator had been expelled (the same site at which it had been inserted). Findings are consistent with the proliferative phase of wound healing, with partial closure, mild erythema, and granulation tissue indicating ongoing epithelialization. Photograph taken on day one of admission.

The patient was afebrile and had stable vital signs and bilateral pitting pedal edema. An approximately 3 cm non-purulent, non-erythematous wound which had healed by secondary intention was visible over the left upper chest (Figure 1). Cardiovascular exam was notable for a systolic murmur but there were no cutaneous signs of infective endocarditis, such as Osler nodes, Janeway lesions, clubbing, or splinter hemorrhages. Examination was otherwise unremarkable.

**Figure 2:**
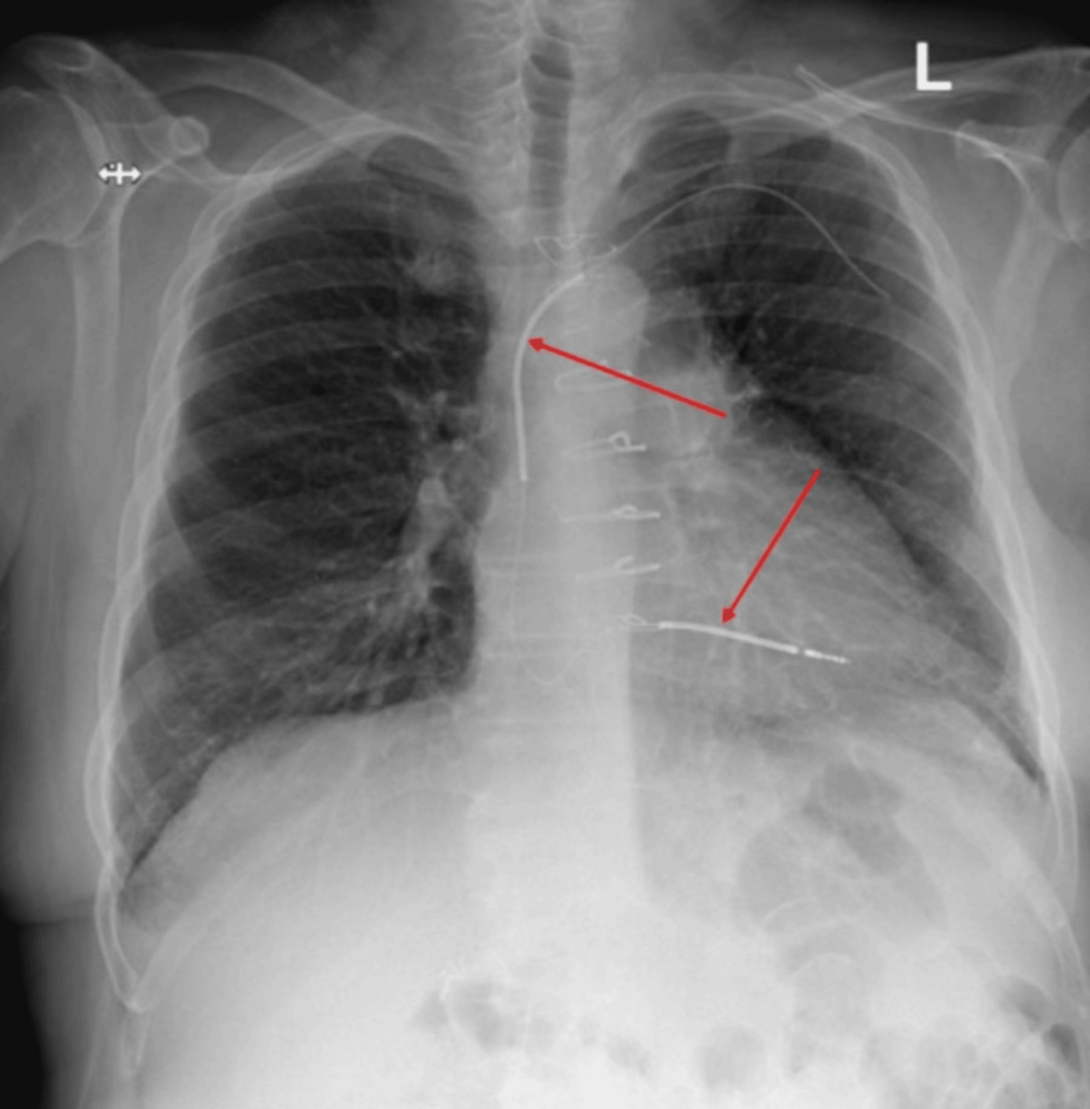
Posterior-Anterior (PA) chest radiograph demonstrating right ventricular implantable cardiac defibrillator lead (red arrows) but missing generator. Chest radiograph taken on day one of admission.

Lab results were notable for a white blood cell count of 12.5 k/mcL (reference range: 4.4-11.3 k/mcL), with an absolute neutrophil count of 10.21 k/mcl (reference range 2.00-9.30 k/mcL). Labs were otherwise unremarkable. C-reactive protein, erythrocyte sedimentation rate, and procalcitonin were not collected. An electrocardiogram showed normal sinus rhythm and a chest radiograph was notable for a right ventricular ICD lead, but showed no other hardware related to the ICD (Figure 2).

**Figure 3:**
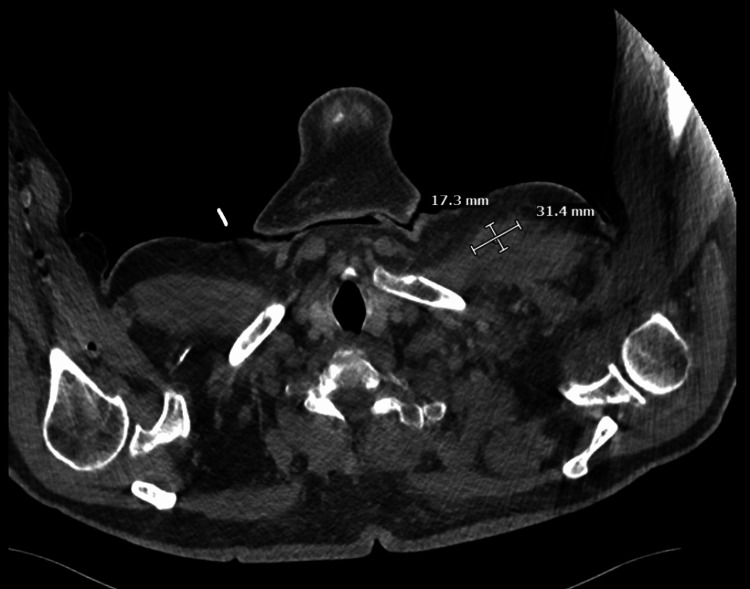
Computed tomography (CT) axial plane imaging of the upper chest demonstrating mild skin thickening and subdermal stranding, with a 1.7 x 3.1 x 2.7 cm underlying fluid collection.

Two sets of blood cultures were taken from different sites on admission and a further two sets were taken approximately 24 hours later. The first two sets of blood cultures were taken five hours following the first dose of intravenous ceftriaxone but before any other antibiotics were administered. One of the aerobic bottles cultured *Staphylococcus hominis* (time to positivity 15.5 hours). A separate aerobic blood culture bottle showed gram-positive rods on Gram stain, which grew *Corynebacterium striatum* on culture (time to positivity four days, 17 hours). A computed tomography (CT) scan of the chest with contrast showed cellulitic changes in the left upper ventral chest, a 1.7 x 3.1 x 2.7 cm fluid collection, and left axillary adenopathy (Figure 3).

The clinical team had a high suspicion for infective endocarditis given the history of cardiac device expulsion and sinus tract creation, recent febrile syndrome, and the retained ICD lead which could act as a foreign body on which bacteria could create a biofilm. 

**Figure 4:**
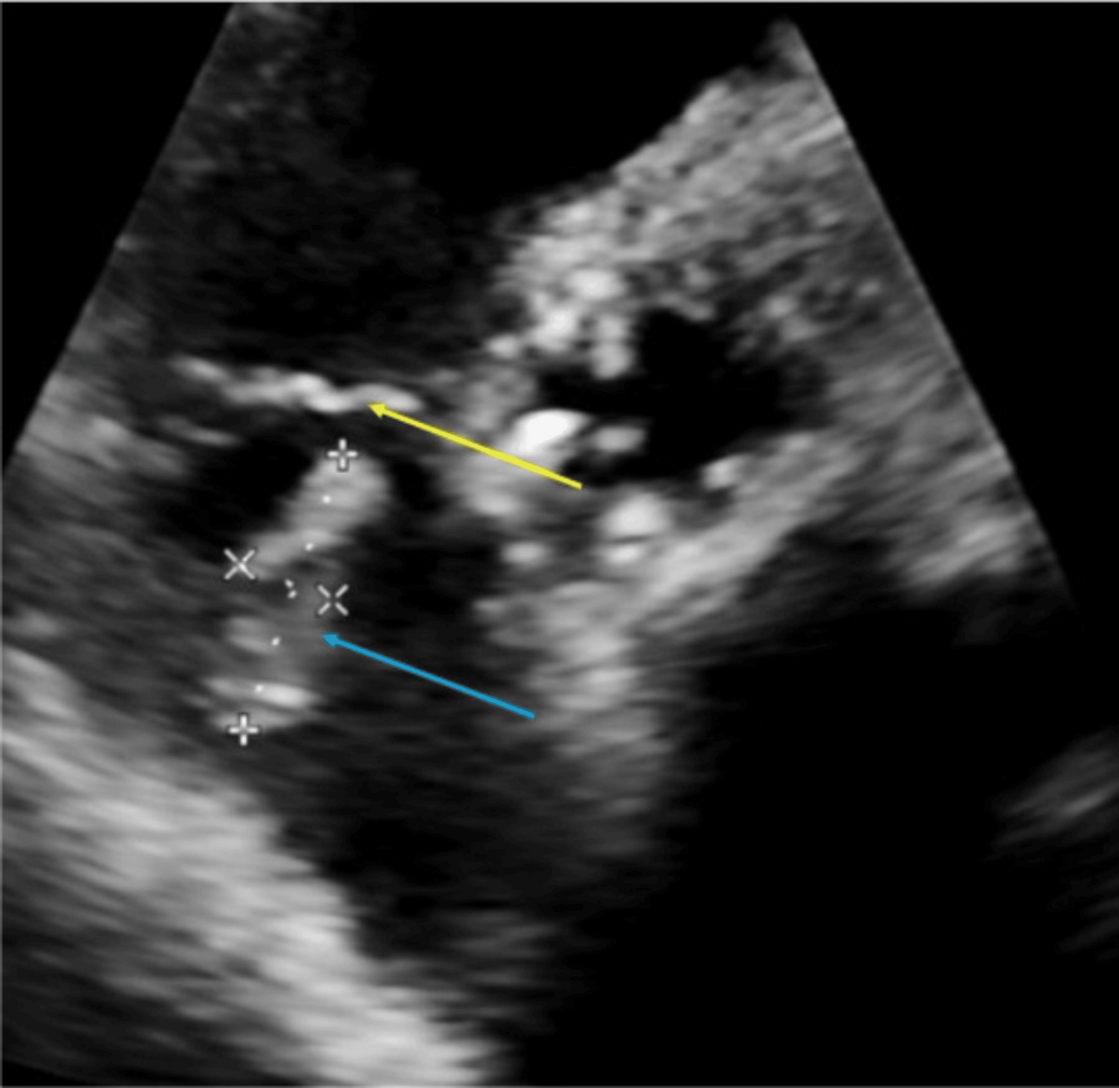
Basal parasternal short axis view from the transthoracic echocardiogram demonstrating the suspected vegetation (blue arrow) and tricuspid valve (yellow). Right ventricle implantable cardiac defibrillator lead not visible in image. Echocardiogram obtained on day three of admission.

A transthoracic echocardiogram was ordered to add evidence to the diagnosis and expedite clinical management. It showed a large, linear, independently mobile echodensity (at least 2.6 x 1.7 cm) which was thought to be on either the atrial surface of the tricuspid valve or the atrial portion of the right ventricular lead, with intermittent prolapse through the tricuspid valve (Figure 4). Mild tricuspid regurgitation was also present. At this point, the patient met Modified Duke’s criteria for infective endocarditis given he had echocardiographic evidence of a vegetation (major criteria), a predisposition to endocarditis given his history of a CIED (minor criteria), fever (minor criteria), and a positive blood culture with a typical organism for infective endocarditis in the setting of intracardiac prosthetic material (minor criteria) [7].

The patient was initially placed on intravenous vancomycin and ceftriaxone for empiric coverage. Three days into admission, he developed a maculopapular rash that was concerning for a drug rash from beta-lactam antibiotics, so ceftriaxone was stopped. Vancomycin was continued as monotherapy given that the *Staphylococcus hominis* was found to be pan-sensitive and it was assumed the *Corynebacterium striatum* would likely be sensitive as well. Cardiology was consulted and decided to forego transesophageal echocardiogram given the high clinical suspicion and positive findings on a transthoracic echocardiogram - instead, they opted to directly pursue percutaneous extraction of the vegetation using the AlphaVac system in order to expedite care. The vegetation was found to be attached to the ICD wire. Following vegetation removal, within the same procedure, the pacemaker pocket was reopened and the ICD wire was removed using a laser to free the lead from the surrounding fibrotic tissue. The pacemaker pocket was also debrided. Tissue from the vegetation and the lead tip were sent for culture. However, no fluid from the pacemaker pocket site was collected for culture - it is unclear whether this was because no fluid was obtained or because this step was missed. The vegetation did not grow any organisms but the ICD lead tip grew both *Corynebacterium striatum* and *Citrobacter koseri*. A summary of all samples sent for culture and their results can be found in Table 1. Given the *Citrobacter* finding, cefepime was added because of initial concerns for Amp-C production which were later not substantiated on antibiotic susceptibility testing. The cefepime did not cause the patient a recurrence of the maculopapular rash he had experienced earlier in the admission. The patient completed a 4.5-week course of cefepime and vancomycin - four weeks from removal of the remaining ICD hardware. He had no recurrence of symptoms and his chest wound continued to heal well.

**Table 1:** Summary of specimens sent for culture ICD: implantable cardiac defibrillator

Day of admission	Specimen source	Timing relative to antibiotics	Culture results	Antibiotic susceptibility testing
1	Blood culture (peripheral)	Taken five hours following first dose of ceftriaxone (prior to all other systemic antibiotics).	Aerobic bottle – *Staphylococcus hominis* (time to positivity 15.5hrs)	Susceptible: oxacillin, vancomycin
			Anerobic bottle – No growth	N/A
1	Blood culture (peripheral)		Aerobic bottle – No growth	N/A
			Anerobic bottle – No growth	N/A
2	Blood culture (peripheral)	Patient had received three doses of vancomycin, two doses of cefepime, and one dose of ceftriaxone prior to culture collection.	Aerobic bottle – *Corynebacterium striatum *(time to positivity 4 days, 17 hours)	Routine susceptibility testing is not performed on Corynebacterium species.
			Anerobic bottle – No growth	N/A
2	Blood culture (peripheral)		Aerobic bottle – No growth	N/A
			Anerobic bottle – No growth	N/A
5	Vegetation tissue	Patient had received 5 days of vancomycin by the time of the procedure, as well as 2 days of ceftriaxone and 1 day of cefepime at the start of the admission.	No growth	N/A
5	Right ventricular ICD Lead		*Corynebacterium striatum* (time to positivity not reported)	Routine susceptibility testing is not performed on Corynebacterium species.
			*Citrobacter koseri* (time to positivity not reported)	Susceptible: amikacin, amoxicillin/clavulanate, ampicillin/sulbactam, aztreonam, cefazolin, cefepime, cefoxitin, ceftriaxone, cefuroxime, gentamicin, piperacillin/tazobactam, tetracycline, tobramycin, trimethoprim/ sulfamethoxazole; Resistant: ampicillin, ciprofloxacin, levofloxacin
N/A	Fluid collection in pocket	Sample not obtained for culture.		

## Discussion

We present a unique case of device-related endocarditis likely resulting from spontaneous expulsion of the device. This led to the creation of a sinus tract from the skin to the heart via the ICD lead, with consequential bacterial seeding and CIED infection. Though rare, such a sinus tract is hypothesized to be a substantial risk factor for endocarditis given the direct communication to the heart and we suggest clinicians maintain a high suspicion for endocarditis early in the clinical course, even in the absence of systemic symptoms. With this patient having a direct tract open from skin to the heart, it is not surprising that he was found to have a polymicrobial endocarditis, growing *Staphylococcal hominis*, *Corynebacterium striatum*, and *Citrobacter koseri*. Given the positive blood cultures for *S. hominis* and *C. striatum*, it is also possible that at least some of these organisms may have spread to the heart via hematogenous seeding from the pocket infection.

We performed a systematic search of EMBASE to identify similar case reports of delayed device expulsion (occurring greater than one month after device implantation) and only found one other case [8]. This occurred in a 70-year-old man who had a permanent pacemaker and had been suffering from one week of chest pain and skin erythema, after which his device ended up eroding through the skin. Blood cultures grew methicillin-resistant *Staphylococcus aureus* (MRSA) and he was treated with device removal, intravenous vancomycin, and mefenamic acid.

Overall CIED-related infections are the most common complications of CIED therapy and have an annual incidence of 2.3 to 3.4% [9]. The risk of infection increases with any subsequent revision or replacement procedure [10]. These infections often have a significant and prolonged impact on a patient’s quality of life, disrupt CIED therapy, and result in long hospital stays with high healthcare costs [11]. Etiologies for CIED-related infections include direct inoculation (which can occur peri-procedurally during primary insertion or revision procedure), skin erosion around the device and its components, or hematogenous spread. Once infection is documented, CIED removal is the mainstay of treatment [6,9] as this complication carries a high mortality rate [12], with incomplete [13] or delayed device removal further worsening prognosis [14]. Percutaneous mechanical aspiration of the vegetation prior to lead extraction is a novel approach in CIED endocarditis that may help minimize the risk of vegetation pulmonary embolization [6]. Other potential benefits include enhancing the antimicrobial therapy response and providing a further opportunity to obtain tissue for microbiological evaluation. In our patient, this was achieved using the AlphaVac system.

Our case’s initial blood cultures were positive for *Staphylococcus hominis*, a coagulase-negative *Staphylococcus* - the group most commonly responsible for CIED infections following *Staphylococcus aureus* [15,16]. Previous reports suggest a significant concordance between lead and blood cultures for staphylococcal infections [15]; however, in our patient, lead cultures did not yield any staphylococcal growth which might have been due to prior receipt of antibiotics. Also of note, the vegetation itself did not culture any bacterial organism, emphasizing the need for a multi-modal diagnostic approach [9]. This may have been the result of prior receipt of several days of antibiotics by the time of aspiration. Nevertheless, given that the organism is commonly implicated in CIED infections [6], it is highly likely that this was a true result and not a blood culture contaminant.

Only 8 to 10% patients have gram-negative and non-staphylococcal CIED infections, and mostly these patients harbor risk factors such as chronic kidney disease, chronic obstructive pulmonary disease, or prior history of device infection [17]. *Corynebacterium striatum* and *Citrobacter koseri* are emerging infectious agents. *Corynebacterium* species are a part of normal skin flora and their growth on blood cultures is often regarded as a contaminant. However, in the latest modified Duke criteria from 2023, *Corynebacterium striatum* and *Corynebacterium jeikeium *are regarded as “typical” pathogens in the context of intracardiac prosthetic material [7]. Therapeutic challenges to controlling *Corynebacterium* infection include its inherent multidrug resistance and its ability to create biofilms which necessitate device removal. There was an initial suspicion that the *Corynebacterium* found in the aerobic blood culture was a contaminant given the prolonged time to positivity. This theory was later placed in doubt as the ICD lead tip also grew *Corynebacterium striatum*, alongside *Citrobacter koseri*. As compared to blood cultures, lead cultures have been found to be more specific [18], thereby guiding the antibiotic therapy in our patient.

*Citrobacter koseri* is a gram-negative commensal whose growth on blood cultures is often addressed aggressively. Previously documented infections included systemic bacteremia, abscesses, and urinary tract infections, but multiple case reports have now found it to be a cause of infective endocarditis as well. In our case, the *Citrobacter* only grew on the lead culture, which may be because of the individual receiving a dose of ceftriaxone five hours before the first set of blood cultures was taken. This underlines the importance of collecting blood cultures prior to antibiotic administration.

## Conclusions

We described an exceptionally rare instance of late, spontaneous ICD expulsion which led to the formation of a sinus tract from the non-sterile skin to the heart and, subsequently, polymicrobial endocarditis. Though rare, device expulsion should be treated seriously with removal of all hardware and evaluation for infective endocarditis, even with minimal systemic symptoms, given its high mortality rate. As we discussed, the diagnosis of polymicrobial endocarditis was evidenced by both the patient meeting Modified Duke’s criteria and the ICD lead that was extracted culturing *Corynebacterium striatum* and *Citrobacter koseri*. Of note, culturing hardware led to highly useful information including the identification of an additional bacterial species as well as improved data on whether to treat the *Corynebacterium striatum* blood culture result as a true result or contamination. We postulate that prompt recognition of device erosion and early and complete system extraction were important in successfully treating the infection. The successful use of percutaneous vegetation removal prior to lead extraction may have helped to mitigate pulmonary embolic risk which could have theoretically occurred as the lead was extracted from the heart. The case illustrates the complexities in tailoring appropriate antibiotic therapy, the diagnostic utility of culturing hardware, and the importance of patient education and close follow-up.
